# The regulatory effects of luteolin, calycosin, and formononetin on the NLRP3/IL-33/ILC2s axis in the treatment of allergic rhinitis: mechanistic analysis and therapeutic potential

**DOI:** 10.3389/fphar.2025.1658772

**Published:** 2025-09-26

**Authors:** Meng Jia, Xiaochun Lei, Fuwei Jiang, Detang Li

**Affiliations:** ^1^ Department of Pharmacy, The First Affiliated Hospital of Guangzhou University of Chinese Medicine, Guangzhou, Guangdong, China; ^2^ Department of Pharmacy, The First Affiliated Hospital of Guangzhou University of Chinese Medicine, Guangzhou, Guangdong, China; ^3^ Department of Pharmacy, Guangdong Clinical Research Academy of Chinese Medicine, Guangzhou, Guangdong, China; ^4^ Department of Pharmacy, Chongqing Hospital of The First Affiliated Hospital of Guangzhou University of Chinese Medicine (Chongqing Beibei Hospital of Traditional Chinese Medicine), Chongqing, China

**Keywords:** allergic rhinitis, luteolin, calycosin, formononetin, NLRP3/IL-33/ILC2s signaling axis

## Abstract

Allergic rhinitis (AR), a common IgE-mediated inflammatory condition of the nasal mucosa, presents with nasal itching, episodic sneezing, and runny nose. Emerging evidence indicates that type 2 innate lymphoid cells (ILC2s) are key players in AR development. Epithelial-derived alarmins (IL-33, IL-25, TSLP) activate ILC2s, leading to Th2 cytokine production (IL-4, IL-5, IL-13) that enhances inflammation. Recent research shows that NOD-like receptor protein 3 (NLRP3) can function as a transcriptional regulator of interleukin-33 (IL-33), offering new mechanistic insights into ILC2s dysregulation. Based on analysis and pharmacological validation of various effective components against AR, three compounds—luteolin, calycosin, and formononetin—have been identified as key ingredients due to their notable anti-inflammatory properties. This review systematically explores how these compounds regulate the NLRP3/IL-33/ILC2s signaling pathway, laying the groundwork for developing targeted AR treatments.

## 1 Introduction

Allergic rhinitis (AR) is one of the most common diseases in otolaryngology, with the incidence rate continuing to rise due to various cultural, economic, and geographical factors. Persistent symptoms such as runny nose, sneezing, nasal congestion, and nasal itching significantly impair patients’ quality of life (Nathan et al., 2008). As a type I hypersensitivity reaction mediated by IgE, AR affects 10%–40% of the global population, placing a significant burden on society and the economy (Zhang et al., 2021a). Recent paradigm shifts emphasize type 2 innate lymphoid cells (ILC2s) as essential innate immune effectors driving AR pathophysiology (Kato, 2019; Luo et al., 2020). Upon allergen exposure, bronchial epithelium-derived alarmins interleukin-33 (IL-33), interleukin-25 (IL-25), and thymic stromal lymphocytin (TSLP) activate ILC2s via ST2 receptors, initiating Th2-polarized immune responses through phosphorylation-dependent activation of NF-κB and MAPK signaling pathways (Hong et al., 2020). The NLRP3 inflammasome, as a standard inflammatory pathway, has been confirmed to play a role in the pathogenesis of AR (Sang et al., 2022). Recent studies have shown that NLRP3 can function as a transcription factor to regulate downstream inflammatory pathways. It can also act as a transcription factor for IL-33 to control the abnormal activation of ILC2s. Furthermore, NLRP3 is involved in the development of AR independently of the NLRP3 inflammasome (Zheng et al., 2021). This review will incorporate the novel idea that NLRP3 acts as a transcriptional regulator in AR through a non-inflammasome pathway. After an initial analysis of the components proven effective against AR, we have discovered that luteolin (Rakariyatham et al., 2018; Hussain et al., 2023; Vajdi et al., 2023), calycosin (Peng et al., 2024), and formononetin (Zhang et al., 2024a; Aladaileh et al., 2019). These three compounds possess strong anti-inflammatory and other pharmacological activities. For example,in a study using an animal model of allergic asthma in mice, calycosin was found to significantly alleviate nasal mucosal inflammation and reduce the levels of Th2-type inflammatory cytokines, such as IL-4, IL-5, and IL-13 (Xue et al., 2021). In addition, luteolin and formononetin also have immunomodulatory effects in various inflammation and immune-related models. Studies have shown that luteolin can reduce the expression of inflammatory factors such as IL-6 and TNF-α by inhibiting the NF-κB and MAPK pathways (Che et al., 2020b). Luteolin has also been shown to alleviate allergic nasal inflammation and inhibit the production of IL-4 in mouse models and peripheral blood mononuclear cells from AR patients (Liang et al., 2020). Formononetin inhibits pro-inflammatory factors and enhances the expression of anti-inflammatory factor IL-10 by regulating the NF-κB and JAK2/STAT3 pathways (Li et al., 2018; Y et al., 2022). Although there is currently a lack of direct evidence of the combined effect of the three monomers in AR models, their independent mechanisms of action support their potential synergistic therapeutic value. Therefore, they can be considered candidate components for treating AR. This review uniquely separates the “nuclear transcriptional function” of NLRP3 from its “cytoplasmic inflammasome function” into two parallel pathways. It systematically compares how three natural compounds intervene selectively at different stages of these pathways, clarifying their complementary mechanisms in treating AR. Due to the side effects associated with hormonal therapy for AR, this review proposes a new therapeutic approach of “non-hormonal, barrier repair first,” encouraging the development of non-hormonal, barrier repair-focused treatments for allergic rhinitis.

In conclusion, this article will examine the potential of the three monomers to influence the NLRP3/IL-33/ILC2s signaling axis, offering a reference for future AR researchers.

## 2 NLRP3 and IL-33 regulatory network

NLRP3 is an important pattern recognition receptor. Its structural domain consists of a N-terminal pyrrole domain (PYD), a nucleotide-binding oligomeric domain (NOD), and a C-terminal leucine-rich repeat (LRR) domain that consists of 12 repeats (Sharif et al., 2019). NLRP3 can function as a transcription factor to activate downstream inflammatory pathways. It can also assemble with ASC and caspase-1 to form the NLRP3 inflammasome. Similar to most inflammasomes, the NLRP3 inflammasome consists of the adaptor protein ASC, PYD, and caspase-1. The ASC adaptor protein includes PYD and CARD domains. The PYD domain of ASC binds to the PYD domain of NLRP3, and the CARD domain of ASC binds to the CARD domain of caspase-1, thereby forming the NLRP3 inflammasome (Xu and Núñez, 2023). Activation of the NLRP3 inflammasome results in the release of pro-inflammatory cytokines, including IL-1β and IL-18, thereby promoting inflammation (He et al., 2016).

Research indicates that NLRP3 can act as a transcription factor to activate inflammatory pathways and influence TH2 differentiation. NLRP3 interacts with IRF4 and enhances the ability of IRF4 and IL4 to bind to and activate the promoter. This suggests that NLRP3 may act as a transcription factor for CD4 Th2 cells (Bruchard et al., 2015). In additionally, NLRP3 also has a regulatory effect on IL-33. Recent studies have shown that NLRP3, localized in the nucleus of epithelial cells, interacts with IRF4 and directly binds to the IL-33-specific promoter, thereby activating its transcription and increasing IL-33 expression. The release of IL-33 further activates downstream signaling pathways, triggering inflammatory responses (Zhou et al., 2024; Im and Ammit, 2014). Notably, a study by Hong et al. (Zheng et al., 2021) demonstrated that in the absence of NLRP3, the expression of IL-33 in airway epithelial cells was reduced by 72.3% (p < 0.001), with significant inhibition of ILC2s activation, leading to reduced production of Th2-type cytokines and emphasizing the critical role of this pathway in AR pathogenesis.

## 3 IL-33/ILC2s/Th2 cascade reaction mechanism

**FIGURE 1:**
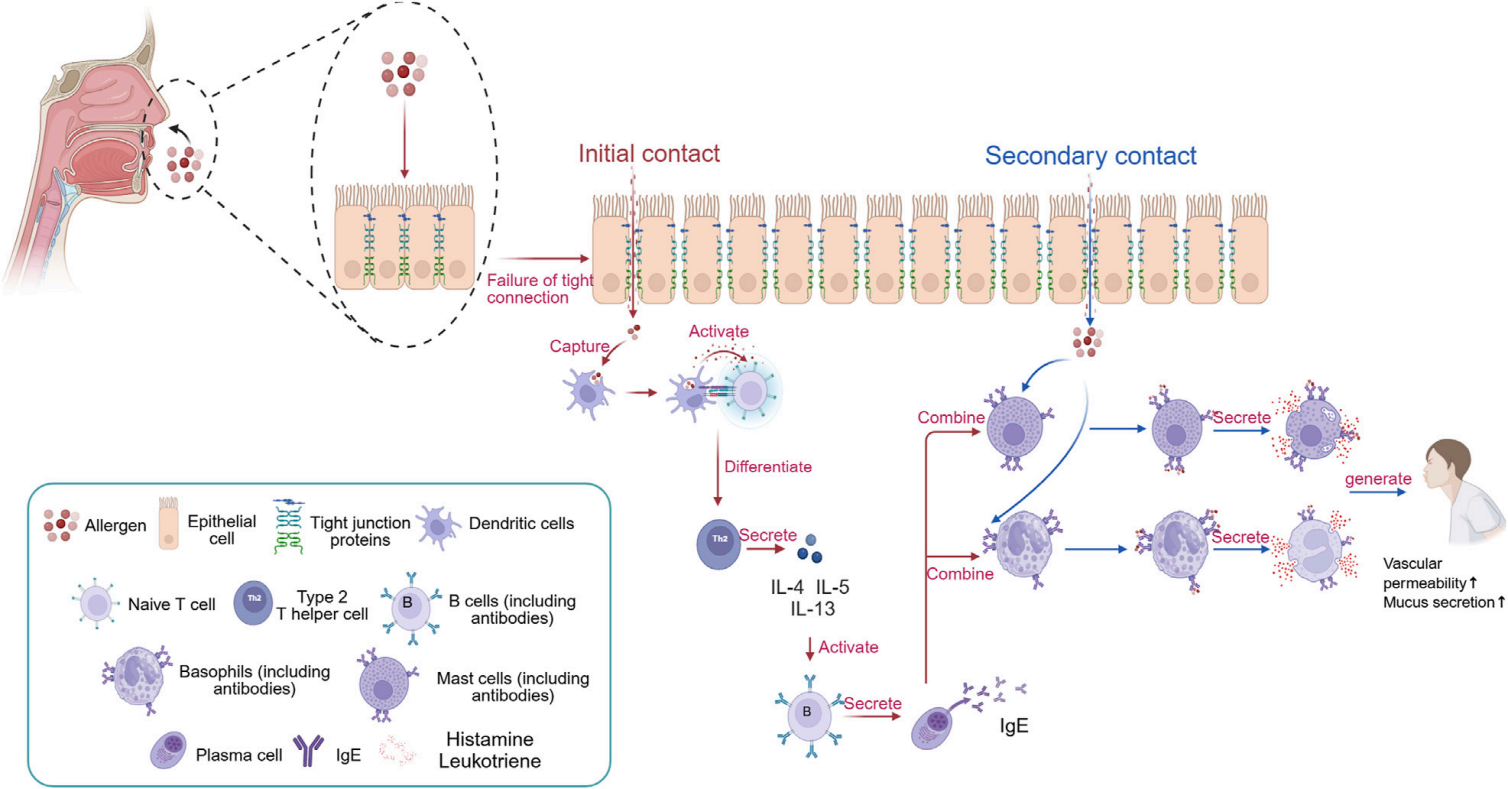
The traditional pathogenesis of AR. When the body first encounters allergens, they disrupt the tight junction proteins of epithelial cells, allowing them to enter the body. At this point, dendritic cells engulf the allergen and degrade it into peptide fragments. Subsequently, dendritic cells transmit signals to naive T cells, causing them to differentiate into Th2 cells. The cytokines produced by Th2 cells (IL-4, IL-5, and IL-13) stimulate B cells to differentiate into plasma cells, which then secrete IgE antibodies. The constant region of IgE antibodies in the body targets mast cells and eosinophils, where they reside in a latent state. When the body is re-exposed to the same allergen, the allergen can specifically bind to the variable region of IgE antibodies on the surface of mast cells and eosinophils. Stimulated mast cells and eosinophils then degranulate, releasing inflammatory mediators such as histamine and leukotrienes, leading to the occurrence of AR. Created in BioRender. xi, l. (2025) https://BioRender.com/pch6h63.

**FIGURE 2:**
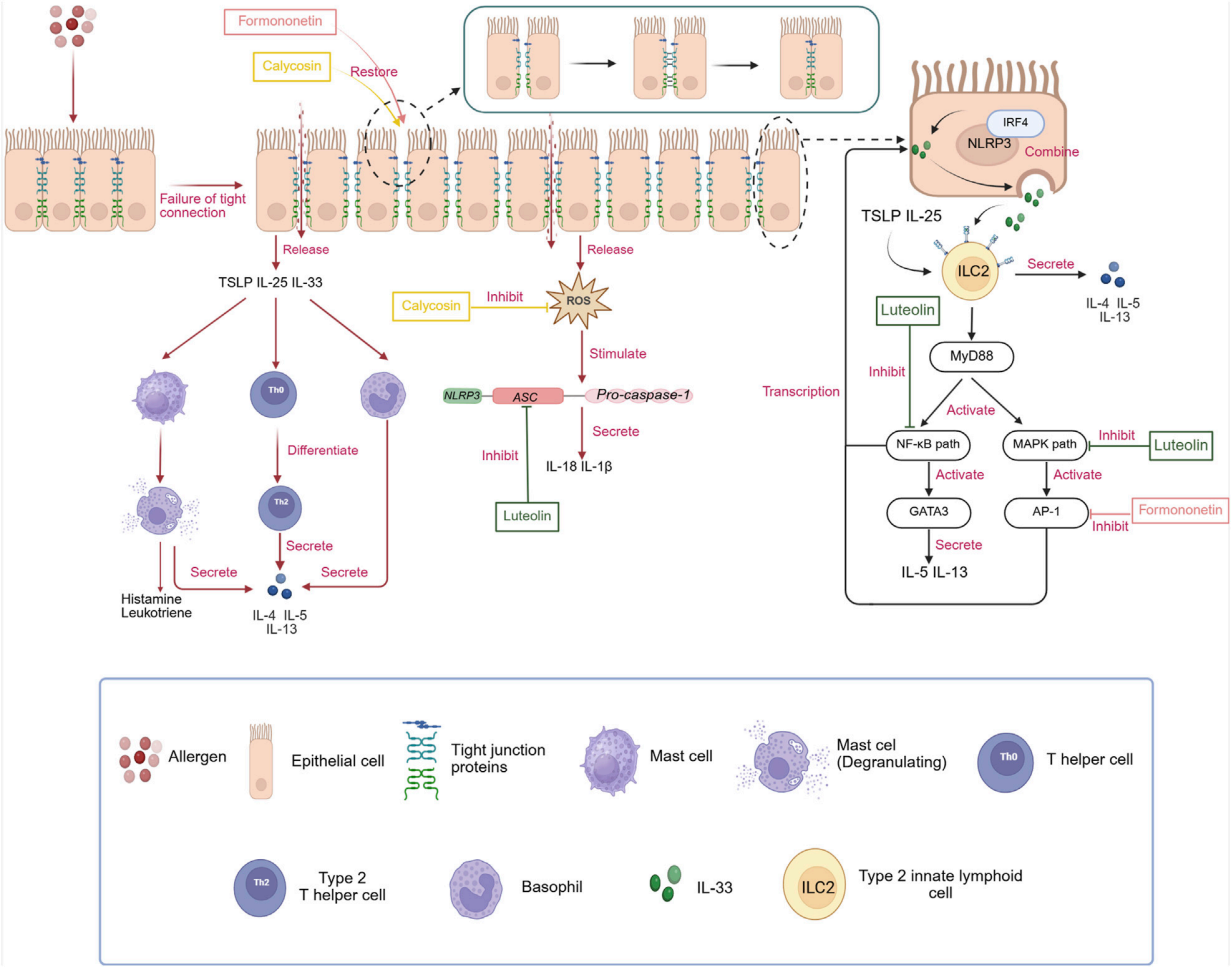
The pathogenesis of AR independent of IgE and the regulatory effects of three monomeric compounds (luteolin, calycosin, and formononetin)on the NLRP3/IL-33/ILC2s pathway in treating AR. i: Unlike the traditional pathogenesis of AR, allergens, after disrupting the tight junction proteins of epithelial cells, cause the release of alarmins, such as TSLP, IL-25, and IL-33, as well as inflammatory substances like ROS. These alarmins directly stimulate the differentiation of Th0 cells into Th2 cells and also directly trigger the secretion of Th2-type cytokines from mast cells and basophils, creating an inflammatory environment. ROS, in turn, directly promote the assembly of downstream NLRP3 inflammasomes, leading to the release of IL-18 and IL-1β, which exacerbate the symptoms of AR. In addition, NLRP3 exists independently of the inflammasome in the nucleus of epithelial cells, interacts with IRF4, binds directly to the IL-33-specific promoter, and activates the transcription of IL-33, increasing its expression. IL-33 released from epithelial cells can also synergistically promote the activation of ILC2 cells with alarmins such as IL-25 and TSLP, leading to a series of subsequent inflammatory reactions. ii: Luteolin can inhibit the activation of the NLRP3 inflammasome by preventing ASC oligomerization, thereby reducing the expression of IL-18 and IL-1β. Furthermore, it can block the MAPK and NF-κB pathways to decrease the transcription of IL-33, thereby regulating the NLRP3/IL-33/ILC2s pathway to treat AR. Calycosin can directly inhibit the generation of ROS, interrupting the signaling for the assembly of the NLRP3 inflammasome. Formononetin can downregulate the expression of the IL-33 transcription factor AP-1 to treat AR. It is worth noting that both calycosin and formononetin can promote the repair of the epithelial cell barrier, fundamentally reducing the stimulation of allergens on epithelial cells and the release of inflammatory factors. Created in BioRender. xi, l. (2025) https://BioRender.com/yxemxgb.

AR is a common allergic disease whose development has long been linked to an imbalance between Th1 and Th2 immunity (Zhang et al., 2021a). However, recent studies have shown that the traditional Th1/Th2 imbalance theory only partially explains the complex immunopathological processes underlying AR. With a deeper understanding of the innate immune system, ILC2s have emerged as key effector cells that initiate early responses in AR (Kato, 2019). Additionally, the essential role of epithelial barrier dysfunction in the development of AR has become increasingly evident (Hellings and Steelant, 2020). Epithelial barrier dysfunction is a key factor in the development of AR. A healthy epithelial barrier can effectively prevent allergens from entering the submucosa of the nasal mucosa, while a damaged barrier allows allergens to penetrate, triggering immune responses and leading to AR. As AR becomes chronic, repeated inflammation continues to damage the epithelial barrier, creating a vicious cycle that results in persistent worsening and chronicity of the condition. Additionally, long-term inflammation can cause apoptosis, necrosis, and other forms of programmed cell death in epithelial cells, further weakening the barrier’s integrity and intensifying the inflammatory response AR (Yuan et al., 2025; Huang et al., 2024). According to the conventional mechanism, when the body is exposed to allergens such as mites, pollen, and dust, the epithelial mucosal barrier is disrupted. This breach greatly increases the likelihood of allergen contact with antigen-presenting cells (APCs), such as dendritic cells, thus amplifying local allergic reactions. Naive T cells, upon recognizing specific antigenic peptides in complex with major histocompatibility complex (MHC) molecules presented by APCs, differentiate into Th2 cells (Breiteneder et al., 2019). These Th2 cells secrete a range of cytokines (IL-4, IL-5, IL-13), which further stimulate B cells to produce allergen-specific IgE. The resulting IgE antibodies then bind to high-affinity IgE receptors (FcεRI) on the surfaces of mast cells and basophils (Chen et al., 2025). Upon re-exposure to the same allergen, it binds to IgE, activating mast cells and basophils (El Ansari et al., 2022). This activation triggers degranulation, releasing histamine, leukotrienes, and other inflammatory mediators that cause allergic symptoms (Figure 1). However, as research deepens, increasing evidence shows that the development of AR can occur independently of IgE (Luo et al., 2020; Hong et al., 2020). Dysfunction of the epithelial barrier directly causes the release of inflammatory cytokines (IL-33, IL-25, TSLP). These cytokines can activate innate immune cells, such as mast cells and basophils, in an IgE-independent manner (Hellings and Steelant, 2020; Kortekaas et al., 2020; Nur Husna et al., 2021; Steelant et al., 2016). IL-33 plays a crucial role in initiating type 2 immunity. The response of ILC2s to IL-33 is significantly more severe compared to that of IL-25. Studies comparing IL-33−/− and IL-25−/− gene mice in airway hyperresponsiveness and acetylcholine-induced airway contraction have shown that diseases stimulated by IL-33 are more severe, with clear activation of ILC2s. This indicates that IL-33 is vital in regulating immune-mediated respiratory responses diseases (Barlow et al., 2013). When allergens breach the epithelial barrier, the release of alarmins like IL-33 activates ILC2s by binding to the ST2 receptor (IL-1RL1) on these cells. This triggers downstream NF-κB and MAPK signaling pathways, leading to high expression of GATA3 and promoting the secretion of Th2-type cytokines ILC2s (Dwyer et al., 2022). Importantly, IL-33 works together with IL-25 and TSLP to boost the response: TSLP increases IL-33 sensitivity by raising ST2 receptor levels on ILC2s, while IL-25 extends ILC2s survival through activation of the STAT5 pathway, creating a positive feedback loop. Activated ILC2s not only release IL-5 and IL-13 directly but also promote Th2 cell polarization via MHC II antigen presentation, enabling communication between adaptive and innate immunity (Hong et al., 2020; Kitano et al., 2022) (Figure 2).

In summary, blocking the activation of ILC2s and their release of Th2-type cytokines is a key strategy for treating AR. Although traditional Chinese medicine (TCM) formulas have proved effective for AR, their complex makeup makes it hard to pinpoint the specific components responsible for their healing effects. This review will, therefore, select effective monomers from TCM formulas used to treat AR based on formula analysis and previous research, to clarify their therapeutic mechanisms.

## 4 Modulation of the NLRP3/IL-33/ILC2s signaling pathway by monomeric compounds

Recent studies have shown that respiratory symptoms such as bronchial asthma, chronic pharyngitis, and allergic rhinitis, which cause coughing, expectoration, and wheezing, are linked to local inflammation infiltration (Eifan and Durham, 2016; Poletti et al., 2006). The main treatment approach for these conditions involves eliminating chronic airway inflammation. Corticosteroids are commonly used in Western medicine to reduce airway inflammation, but their numerous side effects limit their long-term use. According to the literature, 38.3% of patients with allergic rhinitis are refractory to corticosteroids treatment (Sun et al., 2023). In addition, long-term use of corticosteroids may cause weight gain or glaucoma patients (Santiago and da, 2014). Currently, medications are being developed to target type 2 immune responses in AR. Only a few biological agents targeting cytokines such as IL-33 are under development, with most still in phase I or II clinical trials (Hong et al., 2020). Therefore, given the serious treatment situation for AR, it is urgent to find a component with higher safety and fewer side effects to replace it corticosteroids.

Based on statistical analysis, we found that among the TCM with fewer side effects in treating AR, monomeric components such as luteolin, calycosin, and formononetin are particularly significant. The statistical results are as follows (Table 1; Table 2).

**TABLE 1:** TCM formulas containing luteolin for the treatment of AR.

Monomer	TCM containing luteolin	Content level (ug/g)	Key compositional herbs	Chinese medicine combined with Chinese patent medicine to treat AR	Pharmacokinetics
Luteolin	Lonicerae Japonicae Flos (Zhang et al., 2016; Kang et al., 2010; Yang et al., 2025)	19.93–239.49 (Zhang et al., 2024b)	Xanthii Fructus, Magnoliae Flos, Rubiae Radix et Rhizoma, Chrysanthemi Indici Flos	Biyuan Pian	The bioavailability of luteolin in the human body is approximately 25%, with a half-life of 4.5 h. It is mainly metabolized in the liver and excreted through urine (Lee et al., 2021)
			Pogostemonis Herba, Angelicae Dahuricae Radix, Xanthii Fructus, Schizonepetae Herba, et al.	Dare Fragrance Rhinitis Tablets	
	Astragali Radix (Zhang et al., 2022a)	55 (Güven et al., 2023)	Saposhnikoviae Radix, Atractylodis Macrocephalae Rhizoma (fried)	Yu Ping Feng Granule	
			Xanthii Fructus (fried), Saposhnikoviae Radix, Angelicae Dahuricae Radix, Magnoliae Flos et al.	Tongqiao Biyan Capsules	
	Chrysanthemi Flos (Tian et al., 2020; Yang et al., 2024)	205.0–1787 (Wu et al., 2017)	Pogostemonis Herba, Scutellariae Radix, Xanthii Fructus, Ephedrae Herba, et al.	Biyan Kang Tablets	
	Scutellariae Radix (Song et al., 2023; Sonoda et al., 2004)	113.2–17798.8 (Guo et al., 2020)	Asari Radix et Rhizoma, Schizonepetae Herba, Saposhnikoviae Radix, Angelicae Dahuricae Radix, et al.	Xin Qin Granules	

The exact proportions of herbal components in the TCM formulas listed in the table are typically not disclosed in academic literature and are often protected as proprietary intellectual property. The herbal combinations presented under “Key compositional herbs” are all based on their publicly available package inserts, reflecting their main constituent herbs.

**TABLE 2:** TCM formulas containing calycosin and formononetin for the treatment of AR.

Monomer	TCM containing calycosin and formononetin	Content level (ug/g)	Key compositional herbs	Chinese medicine combined with Chinese patent medicine to treat AR	Pharmacokinetics
Calycosin formononetin	Astragali Radix (Cao et al., 2025; Ding et al., 2024; Quan et al., 2015)	Calycosin 35.8–98.5 (Wang et al., 2016)	Saposhnikoviae Radix, Atractylodis Macrocephalae Rhizoma (fried)	Yu Ping Feng Granules	The oral bioavailability of isoflavones is approximately 30%, with a half-life of 5.2 h. Their metabolism mainly involves gluconaldehyde acidification and sulfation reactions (Yuan et al., 2020)
			Xanthii Fructus (fried), Saposhnikoviae Radix, Angelicae Dahuricae Radix, Magnoliae Flos et al.	Tongqiao Biyan Capsules	
			Prunellae Spica, Chrysanthemi Indici Flos, Magnoliae Flos, Saposhnikoviae Radix, et al.	Xiangju Capsules	
		Formononetin 20.32–364.65 (Kim et al., 2007)	Bupleuri Radix, Saposhnikoviae Radix, Rehmanniae Radix (Dried), Mume Fructus, et al.	Jie Min Tang	
	Glycyrrhizae Radix et Rhizoma (Jiang et al., 2022; Li et al., 2024; Wu et al., 2016)	Calycosin 5.93–209.29 (Bo et al., 2002)	Ephedrae Herba, Cinnamomi Ramulus, Paeoniae Radix Alba, Zingiberis Rhizoma, et al.	Xiaoqinglong Granules	The bioavailability of Morinda officinalis is approximately 20%, with a half-life of 3.8 h. The main products after metabolism are excreted through feces (Y et al., 2022)
			Angelicae Dahuricae Radix, Notopterygii Rhizoma et Radix, Asari Radix et Rhizoma, Saposhnikoviae Radix, et al.	Chuan Xiong Cha Tiao Wan	
		Formononetin 110.3–583.68 (Tian et al., 2007)	Magnoliae Flos, Xanthii Fructus (fried), Ephedrae Herba, Angelicae Dahuricae Radix, et al.	Biyan Ning Granules	

The exact proportions of herbal components in the TCM formulas listed in the table are typically not disclosed in academic literature and are often protected as proprietary intellectual property. The herbal combinations presented under “Key compositional herbs” are all based on their publicly available package inserts, reflecting their main constituent herbs.

After conducting a statistical analysis of the effectiveness of TCM components in treating AR, we identified the usage frequencies of three monomers—luteolin, calycosin, and formononetin—in AR research. It is important to note that the analysis of the prescription serves only as a preliminary indication, and final confirmation of the active ingredients still requires further experimental validation. To better understand the therapeutic potential of these TCM monomers, we performed a detailed comparison with conventional drugs (Table 3). The results showed that these TCM monomers have notable advantages in reducing inflammation, repairing nasal mucosal barriers, and lowering recurrence rates, while also exhibiting lower toxicity and fewer side effects. This comparative analysis enhances our overall understanding of the potential of TCM monomers in AR treatment and provides valuable references for future research and clinical application.

**TABLE 3:** AR treatment comparison table: Analysis of the efficacy and safety performance of traditional drugs and Chinese herbal monomers.

Category	Inhibition rate of IL-33	Nasal mucosal barrier repair	Recurrence rate within half a year	Risk of liver toxicity
Corticosteroid	75% (Petersen et al., 2021)	No	42% (Szaleniec et al., 2024)	High (Abdelaziz et al., 2025)
Anti-IgE antibody	28% (McGowa et al., 2023)	No	38% (Hayashi et al., 2025)	Low (Corren et al., 2009)
Luteolin combination	67% (Liang et al., 2020; Hellings and Steelant, 2020)	Significant (Li et al., 2018; Bradding et al., 2024; Yuan et al., 2020)	19% (Xue et al., 2021)	Middle (Aladaileh et al., 2019)

### 4.1 Luteolin: dual inhibition of NLRP3 inflammasome and nuclear transcriptional activation

Luteolin is a natural flavone compound found in various plants. It is generally very safe, with an intraperitoneal LD50 of 411 mg/kg and an oral LD50 of 5,000 mg/kg rats (Xiong et al., 2017). Additionally, it has various pharmacological effects, such as anti-inflammatory, anti-allergic, uric acid-lowering, anti-tumor, antibacterial, and antiviral activities effects (Gendrisch et al., 2021). Studies have demonstrated that luteolin can modulate the NLRP3 inflammasome (Lee et al., 2021), IL-33 and Th2-type cytokines may help treat various diseases. In the progression of AR, damaged epithelial cells release IL-33, which binds to the ST2 receptor on ILC2s, activating them and leading to the secretion of Th2-type cytokines that worsen AR symptoms (Dwyer et al., 2022; Bradding et al., 2024). Research has shown that the release of IL-33 involves complex pathways and substances, with the phosphorylation of AP-1 and NF-κB promoting IL-33 transcription and subsequent release. Luteolin can block the activation of MAPK, NF-κB, and AP-1 pathways (Chen et al., 2024; Zhang et al., 2021b), reducing IL-33 expression and subsequent inflammatory cascades. In allergic diseases, excessive secretion of Th2-type cytokines is viewed as a primary cause. IL-4 is a crucial factor in Th2 cell differentiation, as STAT6 binds to the IL-4 gene promoter to promote its expression and works with GATA3 to enhance Th2 cell differentiation. Luteolin can block the differentiation of Th2 cells by suppressing the IL-4/STAT6/GATA3 signaling pathway, thereby decreasing the secretion of Th2-type cytokines (IL-4, IL-5, and IL-13) and easing allergic symptoms diseases (Chen et al., 2024). Additionally, studies have shown that luteolin can influence the polarization of macrophages from the M1 to the M2 phenotype by downregulating STAT3 and upregulating STAT6, thereby exerting anti-inflammatory effects (Liu et al., 2022). Luteolin not only has strong anti-inflammatory effects but also shows antioxidant activity. In models of myocardial cell inflammatory injury, luteolin lowers reactive oxygen species (ROS) levels and inhibits the activity of the NLRP3 inflammasome (Zhang et al., 2021b). Regarding the assembly of the NLRP3 inflammasome, Lee et al. (2021) demonstrated via cryo-electron microscopy that the ASC oligomer level in the group treated with luteolin was reduced by 67% (*p* < 0.001). Co-immunoprecipitation (Co-IP) showed that luteolin reduced the binding of TXNIP to NLRP3 (binding ↓62%, *p* < 0.01). After dissociating from thioredoxin, TXNIP translocates to the mitochondria and other locations where it binds to NLRP3. ASC, an essential component of the NLRP3 inflammasome, decreased along with the TXNIP-NLRP3 binding, confirming that luteolin reduces the overall level of the NLRP3 inflammasome in the organism. Due to the complexity of biological systems, the interaction between antioxidant and anti-inflammatory mechanisms is complex and bidirectional. The varied pharmacological effects of luteolin provide a strong theoretical basis for developing new therapeutic agents.

### 4.2 Calycosin: modulation of the NLRP3/IL-33/ILC2s signaling pathway

Calycosin, a natural isoflavone compound, shows significant anti-inflammatory and immunomodulatory effects (Deng et al., 2021). In relevant studies, it has been shown to modulate the NLRP3/IL-33/ILC2s signaling pathway (Tian et al., 2024; Liao et al., 2024) and the assembly of the NLRP3 inflammasome (Xia et al., 2021), thereby exerting therapeutic effects. Some studies have shown that in treating intestinal fibrosis, calycosin decreases the mRNA levels of NLRP3 and its downstream caspase-1 and IL-1β in IBD mice, inhibiting the expression of inflammasome-related factors. Additionally, in NLRP3-knockout MODE-K cells, IL-33 signaling is significantly diminished. Therefore, calycosin may have therapeutic potential in intestinal fibrosis by regulating NLRP3 activation and its downstream IL-33/ST2 signaling (Liao et al., 2024). This finding aligns with the idea that NLRP3 can work independently of the NLRP3 inflammasome, laying the groundwork for future research. In cases of ovalbumin-induced allergic asthma, calycosin exerts its therapeutic effect by reducing IL-33/ST2 expression, which inhibits the polarization of ILC2s and M2 macrophages (Tian et al., 2024). Additionally, calycosin plays a role in the assembly and activation of the NLRP3 inflammasome. Xia Y et al. demonstrated that using the DCFH-DA method, calycosin at a concentration of just 20 μM can reduce ROS levels by 78.3% in a sepsis model. Moreover, calycosin dose-dependently inhibits ROS expression, further blocking the assembly of NLRP3, ASC, and pro-caspase-1, reducing the secretion of caspase-1, IL-1β, and IL-18, and thus attenuating the associated inflammatory responses (Xia et al., 2021; Chen et al., 2021). As previously mentioned, during the progression of AR, damaged epithelial cells release IL-33, IL-25, and TSLP, which promote the secretion of Th2-type cytokines by ILC2s, further amplifying the inflammatory response. Calycosin can inhibit the NF-κB pathway, repair epithelial tight junctions, and maintain the integrity of the epithelium barrier (Tao et al., 2017; Yuan et al., 2020), reducing the secretion of IL-33, TSLP, and IL-25 due to epithelial barrier damage, thereby reducing subsequent inflammatory responses. Jia Z et al. (Yuan et al., 2020) demonstrated through Western blot (WB) analysis, that the expression of Occludin protein in HaCaT cells treated with calycosin increased by 2.1-fold (*p* < 0.01). Occludin protein, a crucial component of tight junctions, when missing or impaired, can lead to disruption of intercellular connections, increasing tissue permeability and allowing harmful substances to enter the body through the barrier, potentially triggering diseases. In addition to WB validation showing that calycosin can enhance Occludin protein levels and strengthen epithelial barrier tightness, immunofluorescence (IF) results also indicated that, compared with the modeling group, the tight junctions between cells in the calycosin-treated group were restored in terms of continuity. In allergic asthma models similar to AR, studies have found that calycosin can modulate the activation of ILC2 and M2 macrophage polarization, thereby reducing the secretion of Th2-type cytokines in tissues and achieving therapeutic effects (Tian et al., 2024). From the analysis above, we see that calycosin has unique advantages in the anti-inflammatory process, especially its ability to repair damaged epithelial barriers, maintain barrier function, and reduce the release of related inflammatory factors. This provides a foundation for further exploring calycosin’s pharmacological mechanisms.

### 4.3 Formononetin: modulation of the NLRP3/IL-33/ILC2s signaling pathway

Formononetin, an isoflavone compound found in plants such as Astragalus and Millettia, shows anti-inflammatory and antioxidant activities. Like luteolin and calycosin, formononetin can modulate the expression of the NLRP3 inflammasome, IL-33, and Th2-type responses cytokines (Cho et al., 2019; Jia et al., 2022). In the context of allergic diseases, formononetin has shown significant therapeutic potential. Research indicates that formononetin can downregulate the expression of TSLP and IL-33, thereby helping to restore the epithelial barrier to treat atopic conditions dermatitis (Li et al., 2018) In a middle cerebral artery occlusion model, formononetin inhibits NLRP3 inflammasome activation by suppressing the NF-κB and JAK2/STAT3 signaling pathways, thereby decreasing the secretion of IL-1β and IL-18 (Y et al., 2022). Additionally, Yi et al. (2020) demonstrated via WB that formononetin inhibits JNK phosphorylation (*p* < 0.01). Immunofluorescence colocalization results showed a 63% decrease in nuclear c-Jun content. JNK, as a key MAPK, depends on its phosphorylation for activity, and the nuclear translocation of c-Jun is a crucial step in its function. Formononetin inhibits JNK phosphorylation, thereby decreasing AP-1 activity and preventing its entry into the nucleus, which in turn promotes IL-33 transcription and reduces IL-33 expression. (Yi et al., 2020). Under conditions of epithelial cell damage, formononetin can work synergistically with calycosin to promote cell proliferation and migration, thereby aiding in the repair of the epithelial barrier (Che et al., 2020b). and decreasing the secretion of IL-33, IL-25, and TSLP. In asthma models, groups treated with formononetin showed significantly lower secretion of Th2-type cytokines (IL-4, IL-5, IL-13), demonstrating its strong therapeutic potential in treating allergic diseases. However, the specific pharmacological mechanisms of formononetin need further investigation.

## 5 Discussion and future perspectives

Luteolin exerts strong anti-inflammatory effects by blocking the activation of the NLRP3/IL-33/ILC2s signaling pathway through multiple mechanisms, thereby decreasing the release of inflammatory substances cytokines (Che et al., 2020a; Che et al., 2020b). Its potential therapeutic value in AR has been preliminarily confirmed. Studies have demonstrated that luteolin exerts anti-inflammatory effects by inhibiting the PI3K-Akt signaling pathway (Gao et al., 2023; Chai et al., 2024). However, this pathway is vital for cell growth, proliferation, and survival, and excessive inhibition may cause cell apoptosis and other negative effects. The PI3K-Akt signaling pathway is closely linked to various diseases and is essential for maintaining normal cellular functions. When luteolin inhibits this pathway, it can disrupt the existing signaling balance within cells, leading to halted cell growth and increased apoptosis. Further clinical trials are needed to assess the safety and effectiveness of luteolin. Calycosin, which influences the activation of NLRP3 and its downstream signaling pathways (Zhang et al., 2022b; Chen et al., 2021), suppresses the secretion of related inflammatory cytokines and maintains the integrity of the epithelial barrier (Jia et al., 2018; Yuan et al., 2020), demonstrating significant anti-inflammatory and immunomodulatory effects. Formononetin, like calycosin, promotes the repair of the epithelial barrier (Yuan et al., 2020) and reduces the production of upstream cytokines in AR, thereby alleviating downstream inflammatory responses (Figure 2). The bioavailability of calycosin and formononetin *in vivo* has been shown to be low through chemical analysis and other methods (Lu et al., 2025; Guo et al., 2023; Xiang et al., 2025), which greatly limits their potential for use in treating AR. The absorption efficiency of drugs in the gastrointestinal tract and the strength of the first-pass effect can both cause low effective concentrations in the body, thereby restricting therapeutic effectiveness. To tackle this problem, future research could use nanocarrier delivery (Zou et al., 2024) to enhance the solubility and stability of the drugs, protect them from the harsh gastrointestinal environment, and promote their efficient release at target tissues, thereby improving overall bioavailability and achieving better therapeutic outcomes. After an in-depth investigation into the therapeutic effects of the three monomers on AR, it has been found that these three monomeric compounds exhibit multidimensional synergistic effects in AR treatment: luteolin focuses on inhibiting the signaling pathway (Liang et al., 2020), calycosin enhances barrier function (Jia et al., 2018; Yuan et al., 2020), and formononetin has the advantage of epigenetic regulation. Notably, all three compounds can downregulate IL-33 through different mechanisms (Li et al., 2018; Che et al., 2020b; Elsherbiny et al., 2020). However, their specific sites of action differ, indicating that combination therapy may produce additive effects. Future studies should explore whether the combined use of these compounds can improve therapeutic outcomes. To better illustrate the individual effects of these three monomers in AR treatment, the following table (Table 4) provides a detailed overview of their core structures, binding energies with NLRP3, inhibitory rates on IL-33, and barrier repair efficiencies As illustrated in Table 4, luteolin has the highest binding energy (a measure of interaction strength) with NLRP3 (−8.2 kcal mol^−1^), This pronounced interaction physically occludes the ASC-binding on NLRP3, thereby aborting inflammasome assembly (Lee et al., 2021). This is consistent with its strong inhibitory effect on IL-33 expression (inhibition by 68%). Calycosin, although displaying an intermediate binding energy (−7.5 kcal mol^−1^), demonstrates superior efficacy in restoring epithelial barrier integrity, intercepting the ‘inflammasome–barrier damage–alarmin release’ feed-forward loop at an intermediate step, consequently diminishing IL-33 secretion and indirectly restraining ILC2 activation. Formononetin, possessing the lowest NLRP3-binding energy among the three compounds (−6.9 kcal mol^−1^), appears to operate via an allosteric mode—interfering with AP-1/NF-κB-mediated transcriptional elongation of IL-33 rather than direct NLRP3 engagement—thus exemplifying a ‘weak binding–strong transcriptional repression’ paradigm.

**TABLE 4:** Title:Basic data table of the performance of three monomers in the treatment of AR.

Monomer	Core structure	NLRP3 binding energy (kcal/mol)	Inhibition rate of IL-33 (%)	Barrier repair efficiency
Luteolin	(C2 = C3) (Gendrisch et al., 2021)	−8.2 (Lee et al., 2021)	68% (Che et al., 2020b)	Weak (Bradding et al., 2024)
Calycosin	(C3 = O) (Deng et al., 2021)	−7.5 (Zhang et al., 2022b)	52% (Tian et al., 2024)	Strong (Jia et al., 2018)
Formononetin	(7-OH) (Aladaileh et al., 2019)	−6.9 (Jia et al., 2022)	61% (Yi et al., 2020)	Middle (Li et al., 2018)

The three monomers have complementary characteristics: ‘luteolin targeting intracellular signaling, calycosin strengthening barrier function, and formononetin offering a balanced approach’. These data not only provide experimental evidence for the synergistic effects of the three monomers but also establish a foundation for future research on the potential benefits of their combined use. By examining these specific indicators, we can better understand the unique strengths of each monomer and their potential value in AR treatment.

## 6 Conclusion

The NLRP3/IL-33/ILC2s signaling pathway plays a crucial role in the pathogenesis of AR by activating ILC2s via NLRP3 and cytokines secreted by damaged epithelial cells, thereby promoting the secretion of Th2-type cytokines and exacerbating inflammatory responses. The identification of the NLRP3/IL-33/ILC2s signaling axis provides a novel target for AR treatment. Luteolin, calycosin, and formononetin exert synergistic effects through multi-target and multi-level mechanisms, effectively interrupting the pathological cycle of ‘epithelial damage-alarmin release-ILC2s activation-Th2 polarization’. Compared with traditional corticosteroid therapy, this multi-target regulatory regimen based on natural plant monomers shows significant safety advantages. In light of the limitations of the aforementioned monomers, future research will focus on the development of nanoparticles and nasal spray formulations (Shrestha et al., 2020) to achieve more precise and convenient drug delivery. Meanwhile, on the basis of establishing the synergistic effects of the three monomers, a determination of the synergistic index of the three components will be carried out, and the synergistic effect of combined medication will be verified by the Chou-Talalay method. This will further optimize their ratios and dosing regimens, enabling the drug combination to exert remarkable therapeutic effects at lower doses, thereby enhancing the safety and efficacy of drug therapy as a whole and bringing breakthrough progress to the field of AR treatment.

## Abbreviations

AR, Allergic rhinitis; ILC2s, Type 2 innate lymphoid cells; NLRP3, NOD-like receptor protein 3; IL-33, Interleukin-33; IL-25, Interleukin-25; TSLP, Thymic stromal lymphopoietin; PYD, Pyrin domain; NOD, Nucleotide-binding oligomeric domain; LRR, Leucine-rich repeat; APCs, Antigen-presenting cells; MHC, Major histocompatibility complex; FcεRI, High-affinity IgE receptors; TCM, Traditional Chinese medicine; ROS, Reactive oxygen species; WB, Western blot; IF, Immunofluorescence.
